# Efficacy and safety of deep hyperthermia combined with tislelizumab and chemotherapy in the treatment of advanced squamous non-small-cell lung cancer: a single-center retrospective study

**DOI:** 10.3389/fonc.2026.1770835

**Published:** 2026-04-30

**Authors:** LiYing Guo, Jinshuo Wang, Dongjie Du

**Affiliations:** 1 The third Hospital of Shijiazhuang, The Affiliated Hospital of Hebei Medical University, Shijiazhuang, Hebei, China; 2 Hebei General Hospital Affiliated to Hebei Medicine University, The Affiliated Hospital of Hebei Medical University, Shijiazhuang, Hebei, China

**Keywords:** chemotherapy, deep hyperthermia, efficacy, safety, squamous non-small-cell lung cancer, tislelizumab

## Abstract

**Purpose:**

To investigate the efficacy and safety of deep hyperthermia combined with tislelizumab and chemotherapy in patients with advanced squamous non-small cell lung cancer (NSCLC).

**Methods:**

A total of 100 patients with advanced squamous NSCLC undergoing initial treatment at our hospital between January 2021 and early January 2023 were enrolled. Patients were categorized into an experimental group (n=50) and a control group (n=50). The control group received tislelizumab plus chemotherapy, while the experimental group received deep hyperthermia combined with tislelizumab and chemotherapy. The main objective was objective response rate (ORR). Secondary endpoints encompassed overall survival (OS), progression-free survival (PFS), disease control rate (DCR), and safety.

**Results:**

ORR in the experimental group was 66.0% (33/50), with a DCR of 86.0% (43/50). The ORR and DCR in the control group were 46.0% (23/50) and 84.0% (42/50), respectively. ORR in the experimental group was higher than that in the control group(*P* = 0.044). There was no statistically difference in the incidence of treatment-related adverse events (TRAEs) between the two groups (*P*>0.05). The median PFS and median OS in the experimental group showed no statistically difference compared to the control group (*P* = 0.647; *P* = 0.370).

**Conclusion:**

The combination of deep hyperthermia, tislelizumab, and chemotherapy as an initial therapy for advanced squamous non-small-cell lung cancer (NSCLC) is safe and effective and should be tested on a larger scale in the future.

## Introduction

Lung cancer continues to exhibit a high global incidence rate and remains the leading cause of cancer mortality. Squamous cell carcinoma of the lung constitutes one of the most prevalent pathological subtypes of lung cancer, accounting for approximately 30% of NSCLC (1, 2).Due to its insidious onset, the majority of patients present at an advanced stage, resulting in short survival times and poor prognosis. Chemotherapy remains the standard treatment for advanced NSCLC, though five-year survival rates remain low (3). The advent of immune checkpoint inhibitors has offered new hope for lung cancer patients. The RATIONALE 307 study (4), which evaluated the efficacy and safety of tislelizumab combined with paclitaxel and carboplatin in advanced NSCLC. Results demonstrated superior median PFS in the tislelizumab plus chemotherapy group compared to chemotherapy alone (7.6 months vs 5.5 months, *P* < 0.001).immune checkpoint inhibitors(ICIs) combined with chemotherapy has now become the standard treatment regimen for patients with NSCLC, and the RATIONALE 307 study further confirms the therapeutic role of tislelizumab in this patient population. Tislelizumab possesses a unique humanized immunoglobulin G4 (IgG4) monoclonal antibody targeting programmed cell death 1 (PD-1). It binds specifically to PD-1 with high affinity, minimizing interaction with Fc γ receptors on macrophages. This enables more effective recognition and killing of tumor cells through the mechanism (5). Deep hyperthermia, as one approach to tumor treatment, directly kills tumor cells, enhances sensitivity to radiotherapy and chemotherapy, and inhibits tumor angiogenesis. Concurrently, elevated temperatures boost immune function, stimulate immune cell activity, and amplify immune responses (6, 7). This study employed deep hyperthermia combined with tislelizumab and chemotherapy for advanced squamous cell lung carcinoma, aiming to validate whether this combination therapy demonstrates superior efficacy and safety compared to tislelizumab plus chemotherapy alone.

## Materials and methods

### Patients

**Figure 1:**
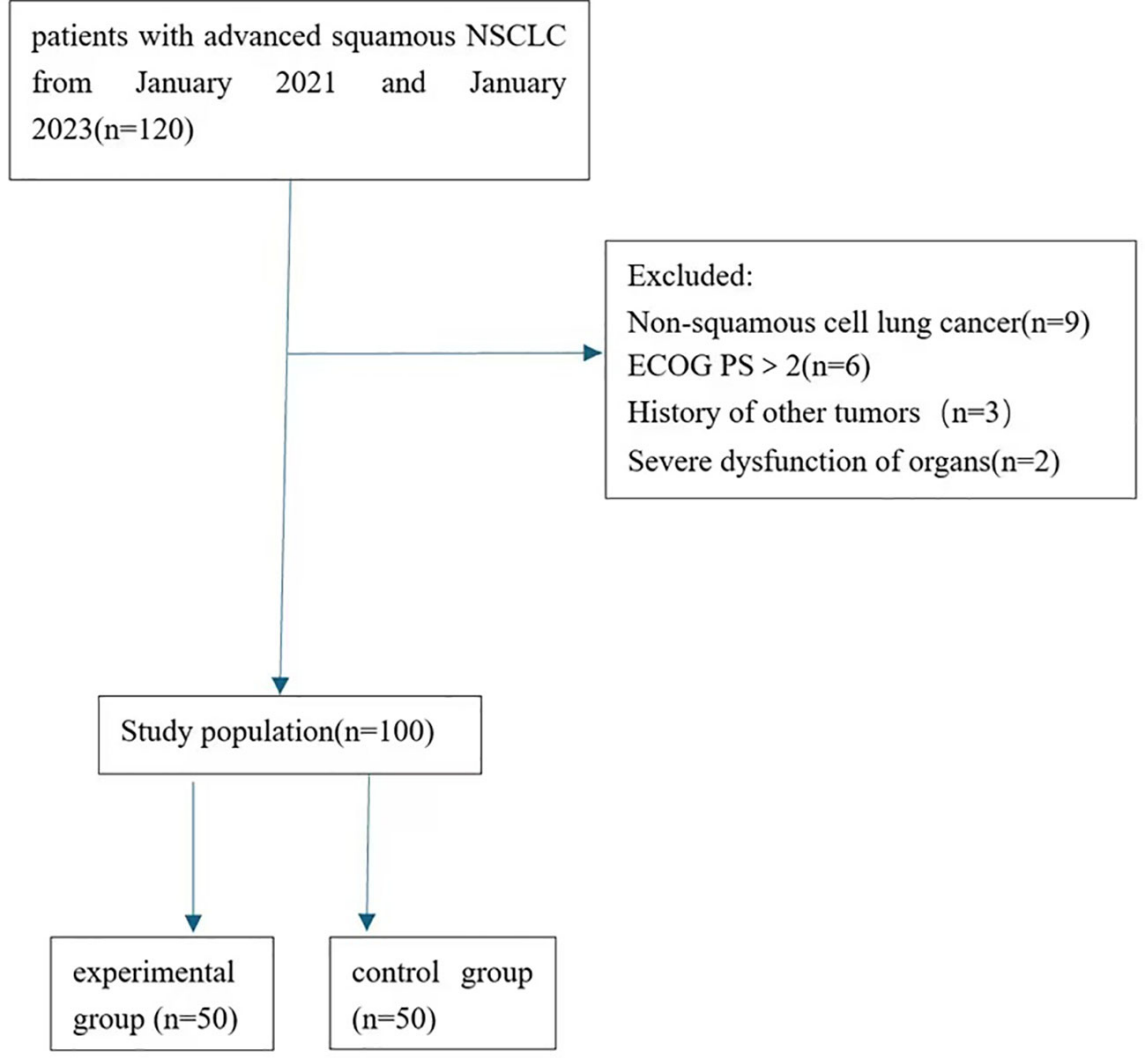
Flowchart of patient selection.

This retrospective study enrolled 100 patients with advanced squamous NSCLC receiving initial treatment in the Third Hospital of Shijiazhuang between January 2021 and January 2023. This study grouped patients based on their actual treatment conditions in previous medical records and whether they were willing to receive deep hyperthermia therapy. Patients with PD-L1 TPS expression level less than 50% were divided into the experimental group and the control group based on whether they received deep hyperthermia treatment, comprising 50 patients each. Patients who received deep hyperthermia therapy and tislelizumab and chemotherapy were classified as the experimental group, while those who received only tislelizumab and chemotherapy were classified as the control group. Detailed data can be found in **Figure 1**. The experimental group included 35 males and 15 females; The median age was 66 years (range: 49–85 years); 37 patients had a history of smoking. The control group comprised 28 males and 22 females; with a median age of 67 years (range: 46–84 years); 27 patients had a history of smoking. There were no statistically significant differences between the two groups in terms of smoking history, age, or disease stage (*P*>0.05), as shown in **Table 1**. Patients were considered eligible for this study if they met the following criteria: (1)Patients with histopathologically or cytologically confirmed inoperable squamous cell lung carcinoma; (2) American Joint Committee on Cancer (AJCC) 8th edition stage IV lung cancer; (3) Eastern Cooperative Oncology Group (ECOG) performance status (PS) score of 0-2; (4) normal function of major organs; (5) life expectancy>4 months;(6) Voluntary signing of informed consent. Ineligible criteria are as follows: (1) History of other tumors; (2) presence of severe organic diseases, immunodeficiency disorders, active viral hepatitis, etc.; (3) severe allergic constitution; (4) psychiatric disorders, non-compliance with treatment or examinations. This study was approved by the Ethics Committee of the Third Hospital of Shijiazhuang, approval number: (2024) Ethics Approval No. 010.

**Table 1:** Patient and tumor characteristics.

Characteristics	The experimental group, n (%)	The control group, n (%)	*χ^2^*	*P*
Sex			2.102	0.147
Male	35 (70.0%)	28 (56.0%)		
Female	15 (30.0%)	22 (44.0%)		
Age			0.040	0.841
>65	27 (54.0%)	26 (52.0%)		
≤65	23 (46.0%)	24 (48.0%)		
Smoking			2.216	0.137
Yes	37 (74.0%)	30 (60.0%)		
No	13 (26.0%)	20 (40.0%)		
ECOG			0.088	0.766
0-1	44 (88.0%)	43 (86.0%)		
2	6 (12.0%)	7 (14.0%)		
Stage			0.161	0.688
IVA	26 (52.0%)	28 (56.0%)		
IVB	24 (48.0%)	22 (44.0%)		
PD-L1 expression in TPS			1.099	0.295
<1%	35 (70.0)	30 (60.0)		
1%-49%	15 (30.0)	20 (40.0)		

### Research methods

The control group received tislelizumab (200mg, day1) combined with nab-paclitaxel and nedaplatin. The specific regimen was as follows: nab-paclitaxel (100mg/m2, days1, 8, and 15), nedaplatin (80 mg/m², day1), with each 21-day period constituting one cycle, totaling four cycles. The experimental group additionally received deep hyperthermia therapy using the HY7000-I model external radiofrequency hyperthermia machine from Nanjing Hengpu Medical Equipment Co., Ltd. The maximum radiofrequency output power was 1500W, with a heating depth of up to 25cm. The treatment target area comprised the primary pulmonary lesion and the cutaneous projection sites of mediastinal lymph node metastases. The target area excludes other metastatic sites. Upper and lower electrode plates and water bags were positioned according to protocol, with the actual treatment temperature in the target area maintained between 40-45 °C. Each session lasted 40 minutes, administered three times weekly. A course comprised four sessions, commencing two hours after chemotherapy or immunotherapy on days when these treatments were administered, and continuing until the conclusion of chemotherapy.

### Evaluation criteria

The key outcome measure was ORR, with additional measures including overall survival (OS), progression-free survival (PFS), disease control rate (DCR) and safety. ORR refers to the percentage of patients achieving a complete response (CR) or a partial response (PR), while DCR refers to the percentage of patients with CR, PR, or stable disease (SD).Researchers assessed target lesions via CT or MRI, evaluating efficacy one month after completion of four treatment cycles according to the Response Evaluation Criteria in Solid Tumors 1.1 (RECIST 1.1); safety was evaluated according to the Common Terminology Criteria for Adverse Events (CTCAE) version 5.0 (8).

### Statistical analyses

SPSS version 26.0 software was used for all statistical analyses. The Chi-square test was used to compare the baseline characteristics between the two groups. the Kaplan–Meier technique was employed to calculate the median duration and 95% confidence interval (*CI*) for PFS and OS. All *P* values are two-sided and *P* values of less than 0.05 were considered to be statistically significant.

## Results

### Efficacy

In the experimental group, the ORR was 66.0% (33/50), and the DCR was 86.0% (43/50). In the control group, the ORR and DCR were 46.0% (23/50) and 84.0% (42/50), respectively. The ORR in the experimental group was higher than that in the control group, with a statistically significant difference (*χ²* = 4.058; *P* = 0.044). There was no significant difference in DCR between the two groups (*χ²* = 0.078; *P* = 0.779) (**Table 2**). The subgroup analysis showed that in patients with PD-L1 TPS expression < 1%, the ORR of the treatment group was 65.7% and the DCR was 85.7%, while the ORR of the control group was 50% and the DCR was 83.3%. There was no statistically significant difference in ORR and DCR (*P* = 0.200; *P* = 0.791). In patients with PD-L1 TPS expression of 1% - 49%, the ORR and DCR of the treatment group were 66.7% and 86.7%, respectively, while those of the control group were 40% and 80%, respectively. There was no statistically significant difference in ORR and DCR (*P* = 0.118; *P* = 0.680) (**Table 3**).

**Table 2:** Tumor response.

Tumor response, n (%)	The experimental group (n = 50)	The control group (n = 50)
Partial response	33 (66)	23 (46)
Stable response	10 (20)	19 (38)
Progressive response	7 (14)	8 (16)
Objective response rate	33 (66)	23 (46)
Disease control rate	43 (86)	42 (84)

**Table 3:** Tumor response by PD-L1 expression.

Tumor response, n (%)	TPS <1%		TPS 1%-49%	
	The experimental group	The control group	The experimental group	The control group
Partial response	23(65.7)	15(50)	10(66.7)	8(40)
Stable response	7(20)	11(36.7)	3(20)	8(40)
Progressive response	5(14.3)	4(13.3)	2(13.3)	4(20)
Objective response rate	23(65.7)	15(50)	10(66.7)	8(40)
Disease control rate	30(85.7)	25(83.3)	13(86.7)	16(80)

### Safety

Prior to immunotherapy and chemotherapy, baseline assessments of patients’ complete blood counts, biochemical parameters, thyroid function, and pituitary and adrenal gland function shall be conducted. During treatment, weekly monitoring of complete blood counts, biochemical parameters, thyroid function, adrenal gland function, and pituitary function shall be performed. Immune-related adverse event(irAE) shall be evaluated every three weeks. Common TRAEs in both groups include leukopenia, thrombocytopenia, and anemia, nausea and vomiting, numbness in hands and feet, fatigue, immune-related hypothyroidism, dermatitis, immune-mediated pneumonia, and immune-mediated enteritis. However, these were predominantly Grade 1–2 adverse events, with Grade 3–4 adverse events occurring infrequently, primarily comprising leukopenia, nausea and vomiting, and anemia. No Grade 3–4 irAE were observed. No hyperthermia-related adverse events such as hypovolemic shock following profuse sweating or burns occurred. The incidence rates of TRAEs between the two groups showed no statistically significant difference (*P* > 0.05) (**Table 4**).

**Table 4:** Common treatment-related adverse events.

Adverse events, n (%)	grade	The experimental group	The control group	*χ^2^*	*P*
Leukopenia	1-2	25 (50.0)	23 (46.0)	0.111	0.739
	3-4	8 (16.0)	6 (12.0)		
Anemia	1-2	30 (60.0)	28 (56.0)	0.165	0.684
	3-4	4 (8.0)	5 (10.0)		
Thrombocytopenia	1-2	15 (30.0)	18 (36.0)	–	1.000
	3-4	0 (0)	1 (2.0)		
Nausea and vomiting	1-2	20 (40.0)	21 (42.0)	0.027	0.869
	3-4	6 (12.0)	7 (14.0)		
Fatigue	1-2	22 (44.0)	25 (50.0)	–	1.000
	3-4	1 (2.0)	2 (4.0)		
Muscular soreness	1-2	10 (20.0)	14 (28.0)	–	1.000
	3-4	0 (0)	1 (2.0)		
numbness in hands and feet	1-2	18 (36.8)	20 (40.0)	–	1.000
	3-4	0 (0)	0 (0)		
hyperthyroidism	1-2	10 (20.0)	9 (18.0)	–	1.000
	3-4	0 (0)	0 (0)		
Immune-mediated skin reaction	1-2	5 (10.0)	3 (6.0)	–	1.000
	3-4	0 (0)	0 (0)		
Immune-mediated pneumonitis	1-2	3 (6.0)	4 (8.0)	–	1.000
	3-4	0 (0)	0 (0)		
Immune-mediated colitis	1-2	2 (4.0)	1 (2.0)	–	1.000
	3-4	0 (0)	0 (0)		

### Survival

**Figure 2:**
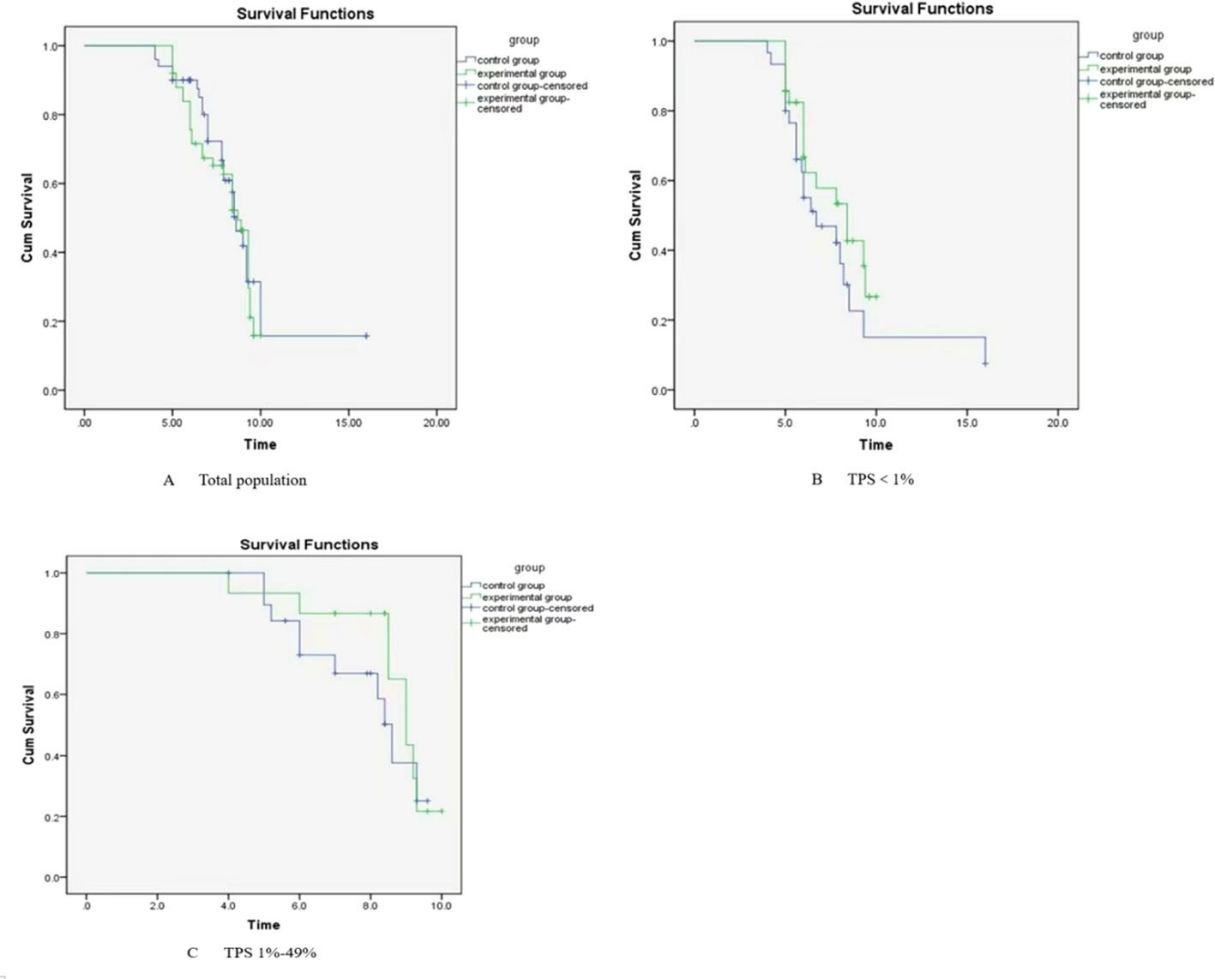
Kaplan-Meier analysis of overall survival (PFS): **(A)** total population, **(B)** <1%, **(C)** 1%-49%.

**Figure 3:**
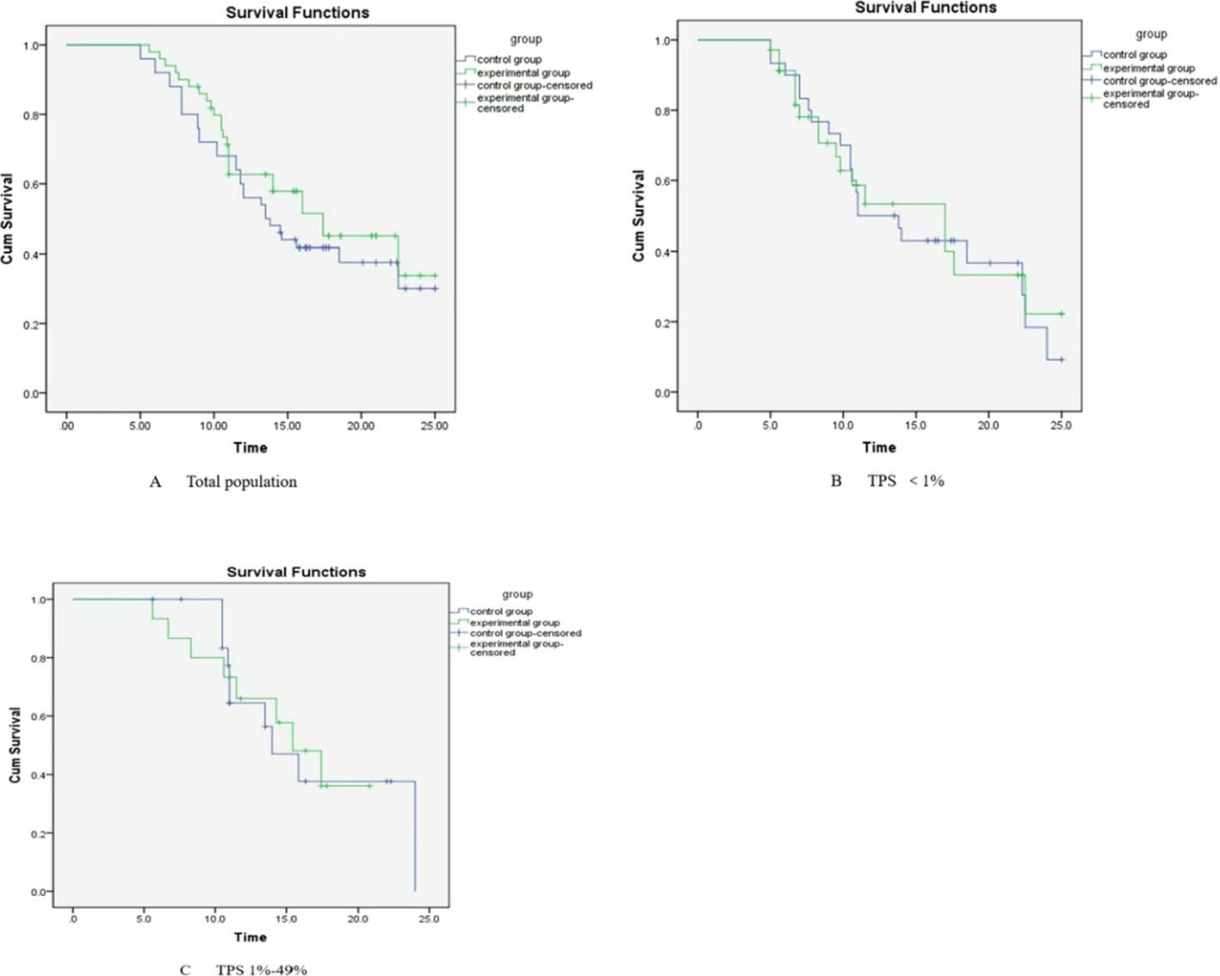
Kaplan-Meier analysis of overall survival (OS): **(A)** total population, **(B)** <1%, **(C)** 1%-49%.

There were a total of 100 patients in the group. None of the patients were lost to follow-up. The follow-up was completed by December 30, 2024. The follow-up period ranged from 5 to 26.6 months, with a median follow-up time of 14.5 months. The median PFS was 8.7 months (95% *CI*: 8.065-9.335) in the experimental group and 8.6 months (95% *CI*: 7.937-9.263) in the control group, showing no statistically significant difference between the two groups (*χ^2^* = 0.209, *P* = 0. 647). The median OS in the experimental and control groups was 17.4 months (95% *CI*: 11.881-22.919) and 13.5 months (95%*CI*: 10.520-16.480), respectively, with no statistically significant difference between the two groups (*χ^2^* = 0.805, *P* = 0.370) 2. The subgroup analysis showed that in patients with PD-L1 TPS expression < 1%, the median PFS of the treatment group was 8.4 months (95% CI: 6.111 - 10.689), while that of the control group was 6.7 months (95% CI: 4.080 - 9.320). There was no statistical significance in the comparison (*χ^2^* = 1.892, *P* = 0.169). The median OS of the treatment group was 17 months (95% CI: 9.572-24.428), and that of the control group was 11 months (95% CI: 6.983-15.017). There was no statistical significance in the comparison (*χ^2^* = 0.051, *P* = 0.822). In patients with PD-L1 TPS expression of 1%-49%, the median PFS of the treatment group and the control group was 9.0 months (95% CI: 8.279-9.721) and 8.6 months (95% CI: 8.049-9.151), respectively. There was no statistical significance in the comparison (*χ^2^* = 0.691, *P* = 0.406). The median OS of the treatment group was 15.4 months (95% CI: 11.428-19.372), and that of the control group was 14 months (95% CI: 10.725-17.275). There was no statistical significance in the comparison (*χ^2^* = 0.009, *P* = 0.925) (**Figures 2**, **3**).

## Discussion

Advanced NSCLC progresses rapidly and exhibits high malignancy. Current conventional treatments include chemotherapy, radiotherapy, and targeted therapy. While platinum-based chemotherapy has historically served as the standard first-line treatment, its efficacy remains limited. With the advent of ICIs, combination ICIs with chemotherapy has now become the standard treatment for patients with advanced squamous NSCLC. Tislelizumab is a humanized monoclonal antibody exhibiting high affinity and specificity for PD-1. Through modification of its Fc segment, it minimizes binding to Fc γ receptors on macrophages, thereby eliminating antibody-dependent cellular phagocytosis (ADCP) (9–11). The RATIONALE 307 (4) is a phase III, randomized controlled trial primarily evaluating the efficacy and safety of tislelizumab combined with paclitaxel/nab-paclitaxel and carboplatin versus paclitaxel and carboplatin as first-line therapy for patients with advanced NSCLC. Results demonstrated median OS of 26.1 months for the tislelizumab plus paclitaxel and carboplatin group, 23.3 months for the tislelizumab plus nab-paclitaxel and carboplatin group, and 19.4 months for the chemotherapy-only group. with corresponding 4-year OS rates of 32.2%, 26.0% and 19.2%. Within the tislelizumab plus chemotherapy group, 42 patients received≥35 cycles of long-term exposure (LTE) to tislelizumab, with this LTE cohort demonstrating a 4-year OS of 97.5%. TRAEs were comparable across all three groups, with most reactions being mild or moderate in severity. The most common TRAEs was hematologic toxicity. This demonstrates that tislelizumab combined with chemotherapy exhibits favorable antitumor activity and durable efficacy in patients with advanced squamous NSCLC (12).

The principle of deep hyperthermia therapy lies in harnessing the biological effects of non-ionizing radiation to destroy tumor tissue or induce tumor cell death. This is achieved by applying physical energy to heat specific areas or the entire body, utilizing the resulting secondary effects to elevate tumor tissue temperature to a specific therapeutic level and maintain it for a sustained period. By exploiting the differing thermal tolerance between normal cells and tumor cells, the aim is to eliminate tumor cells while sparing surrounding healthy tissue. Combining hyperthermia with ICIs can influence the quantity and function of immune cells such as T cells and NK cells, regulate the expression of immune-related cytokines, and enhance the body’s immune capacity. Simultaneously, it can further impact the tumor immune microenvironment by promoting the production of heat shock proteins, thereby improving the body’s anti-tumor capabilities (13–15). Ohta et al. (16) investigated the effects of mild hyperthermia on the tumor microenvironment (TME) of squamous cell carcinoma using a mouse model. Results demonstrated increased PD-L1 mRNA levels and surface PD-L1 expression following mild hyperthermia, enhancing the accumulation of CD4+ and CD8+ T cells and elevating PD-L1 expression within the TME. Takeda et al. (17) enrolled 3,419 patients with advanced or recurrent cancer treated between June 2005 and December 2023. Among these 2,329 patients received dendritic cell therapy, achieving an overall response rate of 25.4%. Among 140 patients receiving combined dendritic cell therapy and ICI treatment, the response rate reached 56.4%. Adding hyperthermia elevated the response rate from 40.0% to 57.7%. Concurrently, this study demonstrated that elevated temperatures promote immune cell infiltration and increase PD-L1 expression. Xiao Jing et al. (18) analyzed 84 patients with advanced squamous NCSLC who were negative for driver gene mutations and positive for PD-L1 expression. They were divided into the experimental group and a control group. The control group received sintilimab combined with nab-paclitaxel and carboplatin, while the experimental group received the same treatment plus deep thoracic hyperthermia. Results demonstrated an ORR of 71.43% in the experimental group, significantly higher than the 50% observed in the control group (*P* = 0.044). DCR showed no statistically significant difference (*P* = 0.212). Common TRAEs in both groups included thrombocytopenia, neutropenia, leukopenia, anemia, and fatigue, predominantly grades 1-2. The incidence rates of adverse reactions between groups were not statistically significant (*P*>0.05). The median OS in the experimental group was 12.9 months, compared with 9.7 months in the control group, representing a statistically significant difference (*P* = 0.035). The ORR in the experimental group was 68.6%, with DCR of 88.6%. The corresponding rates for the control group were 42.9% and 80.0%, respectively. The ORR in the experimental group was higher than that in the control group, with a statistically significant difference (*χ^2^* = 4.690, *P* = 0.030). There was no significant difference in DCR between the two groups (*χ^2^* = 0.97, *P* = 0.324). The incidence rates of adverse reactions in both groups did not differ significantly (*P >*0.05). Consistent with findings by Xiao Jing et al. (18), the improved ORR suggests hyperthermia may enhance tumor response. However, this study found no significant difference in median PFS or median OS between the treatment and control groups (*P* = 0.414; *P* = 0.512), indicating that the addition of deep hyperthermia does not conclusively prolong long-term patient survival.

We all know that patients with high PD-L1 expression can significantly benefit from immune checkpoint inhibitors. However, in the distribution of the Chinese population, the proportion of patients with high PD-L1 expression is relatively small. Therefore, this study included patients with PD-L1 expression less than 50%.The subgroup analysis of this study showed that regardless of the PD-L1 TPS expression level, there was no significant difference in the ORR and DCR between the two groups (*P* > 0.05), and the median PFS and median OS in the experimental group were not significantly different from those in the control group (*P* = 0.414; *P* = 0.512). This was considered due to the fact that a larger proportion of PD-L1 TPS low-expression individuals were included in this study, resulting in a relatively lower responsiveness to immune checkpoint inhibitors and deep hyperthermia treatment compared to the high-expression individuals. Moreover, the sample size of this study was insufficient, which led to a decline in the ability to detect true differences and prevented reaching statistical significance. The insufficient follow-up time prevented the significant detection of the benefits of PFS and OS, and there might be heterogeneity among the included patient populations. Some patients may be sensitive to the drugs, resulting in an increase in ORR, but the overall population may have patients with resistance or poorer prognosis, preventing the overall benefits of PFS and OS from being reflected.

Currently, deep hyperthermia and ICIs are widely employed in clinical practice, whilst the combination of hyperthermia with ICIs represents a novel therapeutic frontier, its advancement underpinned by the concurrent development of both hyperthermia and ICIs. Regarding ICIs, we must further identify new biomarkers, explore new pharmaceutical agents, enhance patient efficacy whilst mitigating adverse reactions. Regarding deep hyperthermia, precision hyperthermia and precise temperature control represent areas requiring further investigation. Naturally, this study has limitations: the small sample size precluded subgroup analyses, making it impossible to rule out confounding factors influencing the results. The limited sample size may have affected the statistical significance of PFS/OS, and the study featured a single pathological type with relatively short follow-up periods. Subsequent studies will optimize hyperthermia parameters, expand sample sizes, and conduct analyses targeting PD-L1 positive populations. Substantial foundational research and clinical trials remain essential, with hopes that large-scale studies will bring renewed hope to more advanced cancer patients.

## Conclusion

In summary, the combination of deep hyperthermia and tislelizumab plus chemotherapy demonstrated promising antineoplastic efficacy and a manageable safety profile in advanced squamous NSCLC patients.

## Data Availability

The original contributions presented in the study are included in the article/supplementary material. Further inquiries can be directed to the corresponding author.
